# Polyamine Biosynthetic Pathway as a Drug Target for Osteosarcoma Therapy

**DOI:** 10.3390/medsci6030065

**Published:** 2018-08-16

**Authors:** Rebecca R. Weicht, Chad R. Schultz, Dirk Geerts, Katie L. Uhl, André S. Bachmann

**Affiliations:** 1 Department of Pediatrics and Human Development, College of Human Medicine, Michigan State University, 400 Monroe Avenue, NW, Grand Rapids, MI 49503, USA; weichtgr@msu.edu (R.R.W.); Chad.Schultz@hc.msu.edu (C.R.S.); Katie.Uhl@hc.msu.edu (K.L.U.); 2 Helen DeVos Children’s Hospital, Department of Pediatric Hematology Oncology, Grand Rapids, MI 49503, USA; 3 Department of Medical Biology, Amsterdam University Medical Center, University of Amsterdam, 1105 AZ Amsterdam, The Netherlands; h.a.geerts@amc.uva.nl

**Keywords:** cell differentiation, DFMO, ornithine decarboxylase, osteosarcoma, polyamines

## Abstract

Osteosarcoma (OS) is the most common bone tumor in children. Polyamines (PAs) are ubiquitous cations involved in many cell processes including tumor development, invasion and metastasis. In other pediatric cancer models, inhibition of the PA biosynthesis pathway with ornithine decarboxylase (ODC) inhibitor alpha-difluoromethylornithine (DFMO) results in decreased cell proliferation and differentiation. In OS, the PA pathway has not been evaluated. DFMO is an attractive, orally administered drug, is well tolerated, can be given for prolonged periods, and is already used in pediatric patients. Three OS cell lines were used to study the cellular effects of PA inhibition with DFMO: MG-63, U-2 OS and Saos-2. Effects on proliferation were analyzed by cell count, flow cytometry-based cell cycle analysis and RealTime-Glo™ MT Cell Viability assays. Intracellular PA levels were measured with high-performance liquid chromatography (HPLC). Western blot analysis was used to evaluate cell differentiation. DFMO exposure resulted in significantly decreased cell proliferation in all cell lines. After treatment, intracellular spermidine levels were drastically decreased. Cell cycle arrest at G_2_/M was observed in U-2 OS and Saos-2. Cell differentiation was most prominent in MG-63 and U-2 OS as determined by increases in the terminal differentiation markers osteopontin and collagen 1a1. Cell proliferation continued to be suppressed for several days after removal of DFMO. Based on our findings, DFMO is a promising new adjunct to current osteosarcoma therapy in patients at high risk of relapse, such as those with poor necrosis at resection or those with metastatic or recurrent osteosarcoma. It is a well-tolerated oral drug that is currently in phase II clinical trials in pediatric neuroblastoma patients as a maintenance therapy. The same type of regimen may also improve outcomes in osteosarcoma patients in whom there have been essentially no medical advances in the last 30 years.

## 1. Introduction

Osteosarcoma (OS) is the most common bone tumor in children, with approximately 400 children diagnosed annually in the United States. With our current treatments we have achieved approximately 70% cure rates for patients presenting with localized disease. However, OS is often metastatic at diagnosis and only about 30% of children survive in this scenario. Treatment of OS involves a combination of aggressive chemotherapy and surgery [1,2]. Unfortunately, there have not been any significant advances in OS treatment or outcomes since the 1980s [3]. 

Polyamines (PAs) are small molecules found in all cells [4,5,6]. They participate in many cell processes including angiogenesis, immune regulation, cell growth, cell signaling and apoptosis [7,8,9]. They are also known to be involved in tumor development, invasion and metastasis [10,11,12,13,14,15,16]. Polyamines are absorbed from the diet and intrinsically produced. Ornithine decarboxylase (ODC) is a rate-limiting enzyme in PA biosynthesis [10,17]. Alpha-difluoromethylornithine (DFMO) blocks PA synthesis by inhibiting ODC [10,11,15,18,19]. DFMO has been evaluated for both treatment and chemoprevention in a number of adult cancers with promising outcomes [15,19,20,21,22,23,24]. Its investigation in pediatric cancers has largely been limited to neuroblastoma, in which PA depletion resulted in G_1_ cell cycle arrest and differentiation [10,14,16,25,26,27]. These findings led to several clinical trials which show promising results [28]. 

In OS, the role of PAs and the effect of DFMO have not been evaluated. However, PAs are known to be involved in osteogenic differentiation in a complex way. Some studies have shown that exogenous PAs stimulate osteogenic differentiation, while others have shown that polyamine depletion, via ODC inhibition with DFMO, promotes osteogenic differentiation of mesenchymal stem cells [29,30,31]. In this study we found that PA depletion with DFMO in OS cell lines resulted in decreased cell viability and differentiation. Remarkably, the effects of DFMO were persistent even after removal of the drug. Our results suggest that PA biosynthesis plays an important role in OS and that the targeting of this pathway may have clinically significant effects.

## 2. Materials and Methods

### 2.1. Chemicals, Reagents and Antibodies

The ODC inhibitor DFMO was provided by Dr. Patrick Woster (Medical University of South Carolina, Charleston, SC, USA). Dansylated spermidine and 1,7-diaminoheptane standards were provided by Dr. Otto Phanstiel (University of Central Florida, Orlando, FL, USA). High-performance liquid chromatography (HPLC)-grade methanol, HPLC-grade acetonitrile, and methylene chloride were obtained from Fisher Scientific (Hampton, NH, USA). Rabbit monoclonal glyceraldehyde 3-phosphate dehydrogenase (GAPDH) antibodies were obtained from GeneTex (Irvine, CA, USA). Mouse monoclonal antibodies against osteopontin (OPN), GAPDH, and collagen 1a1 (Col1a1) were obtained from Santa Cruz Biotechnology (Dallas, TX, USA). Rabbit monoclonal antibodies against alkaline phosphatase were obtained from Abcam (Cambridge, MA, USA). RealTime-Glo MT Cell Viability Assay was obtained from Promega (Madison, WI, USA). Goat anti-mouse or anti-rabbit secondary antibodies conjugated to IRDye^®^ 680RD or IRDye^®^ 800CW were obtained from Licor (Lincoln, NE, USA). Protein assay dye reagent was obtained from Bio-Rad Laboratories (Hercules, CA, USA). 

### 2.2. Culturing of Osteosarcoma Cells

The human OS cell lines MG-63 (CRL-1427), U-2 OS (HTB-96), and Saos-2 (HTB-85) were cultured in Roswell Park Memorial Institute (RPMI) 1640 media (VWR, Radnor, PA, USA), supplemented with 10% heat-inactivated fetal bovine serum (FBS) (Invitrogen, Carlsbad, CA, USA), penicillin (100 IU/mL) and streptomycin (100 μg/mL) (Corning, Corning, NY, USA). The three OS cell lines which express c-MYC and ODC (Supplementary Figure S1) were purchased from the American Type Culture Collection (ATCC, Manassas, VA, USA) within the last two years. The cells were maintained at 37 °C in a humidified atmosphere containing 5% CO_2_. For DFMO treatments, cells were plated, allowed to settle overnight, and then exposed to 5 mM DFMO for six days. DFMO and/or media of cells were replaced on day 3.

### 2.3. Cell Viability

Cell viability assays were performed using the RealTime-Glo™ MT Cell Viability Assay according to the manufacturer’s protocol (Promega, Madison, WI, USA). After cells had been exposed to 5 mM DFMO for six days they were reseeded in standard media in white-walled 96-well plates at a density of 2000 cells/well (MG-63) or 4000 cells/well (U-2 OS and Saos-2). Cells were allowed to attach overnight. For time-zero measurements, cells were incubated with RealTime-Glo™ MT Cell Viability reagent for 20 min at 37 °C, and luminescence was measured on a Synergy (Biotek, Winooski, VT, USA) microplate reader. Luminescence was then measured at 24, 48, and 72 h after the addition of RealTime-Glo™ reagent.

### 2.4. Western Blot Analysis

Whole cell lysates were prepared using radioimmunoprecipitation assay (RIPA) buffer (20 mM Tris-HCl (pH 7.5), 135 mM NaCl, 2 mM ethylenediaminetetraacetic acid (EDTA), 0.1% (w/v) sodium lauryl sulfate, 10% (v/v) glycerol, 0.5% (w/v) sodium deoxycholate, and 1% (v/v) Triton X-100). The RIPA buffer was supplemented with cOmplete™ Protease Inhibitor Cocktail (Roche, Basel, Switzerland), and 0.27 mM Na_3_VO_4_ and 20 mM NaF as phosphatase inhibitors. Protein concentration was determined using the Bradford dye reagent protein assay (Bio-Rad, Hercules, CA, USA). Cell lysates in sodium dodecyl sulfate (SDS) sample buffer were boiled for 5 min and equal amounts of protein were resolved by 10% or 12% SDS–polyacrylamide gel electrophoresis (PAGE). Protein was electrotransferred onto 0.45 µM polyvinylidene difluoride Immobilon-P membrane (Millipore, Burlington, MA, USA). Primary antibodies were incubated overnight at 4 °C in 5% bovine serum albumin (BSA) in Tris-buffered saline containing 0.1% Tween-20. Secondary antibodies were incubated for up to 2 h at room temperature in Tris-buffered saline containing 0.1% Tween-20. Blots were imaged using an Odyssey Fc or Odyssey CLx (Licor, Lincoln, NE, USA) Western blot scanner. Western blot quantitation was performed using Image Studio Lite version 5.2 (Licor).

### 2.5. Measurement of Polyamines

Polyamines from treated OS cells were isolated, dansylated, and analyzed by HPLC as previously described [32,33]. Briefly, PAs were extracted and protonated in perchloric acid/sodium chloride buffer. To 100 µL of sample, 4.5 nmol of 1,7-diaminoheptane internal standard and 200 μL of 1 M sodium carbonate were added prior to dansylation with 400 µL of 5 mg/mL dansyl chloride (Sigma-Aldrich, St. Louis, MO, USA). Samples were analyzed using a Thermo Scientific/Dionex Ultimate 3000 HPLC (Thermo Scientific, Waltham, MA, USA) equipped with a Syncronis C18 column (250 × 4.6 mm, 5 µM pore size). The dansylated PA derivatives were visualized by excitation at 340 nm and emission at 515 nm. Using the relative molar response derived from *N*-dansylated PAs and 1,7-diaminoheptane standards, the amount of *N*-dansylated PAs was calculated and normalized to total sample protein.

### 2.6. Flow Cytometry Cell Cycle Analysis

Cells were collected, fixed in 70% cold ethanol overnight and stained with phosphate-buffered saline (PBS) containing 50 μg/mL propidium iodide and 100 ug/mL RNase A for 2 h at 37 °C. Cells were subjected to flow-cytometric analysis using a Cytoflex S flow cytometry instrument (Beckman Coulter, Miami, FL, USA). Cell cycle distribution was determined using the ModFit software (Verity Software House, Topsham, ME, USA).

### 2.7. Statistical Analyses

The statistical significance of DFMO treatment in cell viability experiments and polyamine analysis was determined using an unpaired Student’s *t*-test assuming the null hypothesis. For all tests, a value of *p* < 0.05 was considered statistically significant. 

Additional materials and methods are provided under Supplementary Information.

## 3. Results

### 3.1. Alpha-difluoromethylornithine Treatment Decreases Osteosarcoma Cell Proliferation

**Figure 1:**
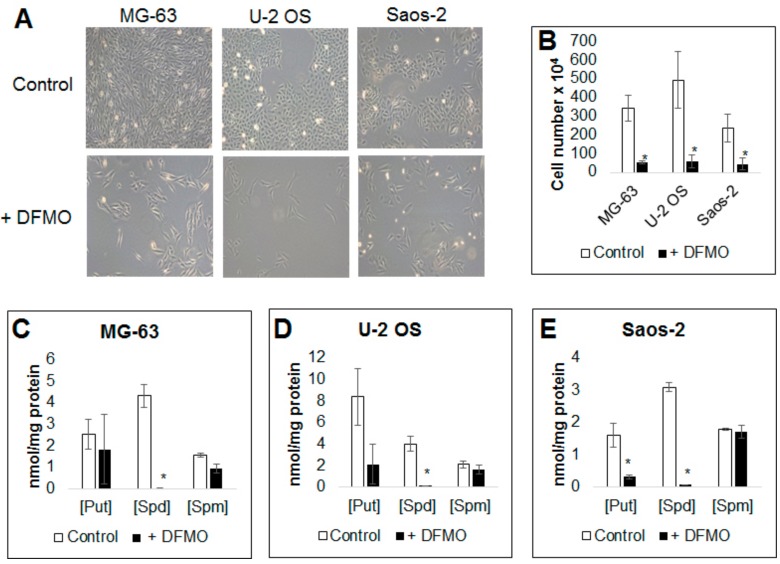
Effects of polyamine inhibitor alpha-difluoromethylornithine (DFMO) on tumor cell growth and polyamine profile of human osteosarcoma (OS) cells. (**A**) Representative light micrographs of MG-63, U-2 OS and Saos-2 cells grown in the presence or absence of 5 mM DFMO for six days. Light micrographs were routinely taken with a Leica (Wetzlar, Germany) DMi1 light microscope to document most of the experiments throughout this study and the pictures are representative of 5 separate experiments (*N* = 5); (**B**) viable cells were counted with a hemocytometer and trypan blue after 6 days of 5 mM DFMO exposure. DFMO treatment drastically reduced cell number in all cell lines; (**C**–**E**) levels of intracellular polyamines putrescine (Put), spermidine (Spd) and spermine (Spm) were measured by high-performance liquid chromatography (HPLC) in OS cells after exposure to 5 mM DFMO for six days. DFMO treatment resulted in a consistent decrease of putrescine and spermidine levels in all cell lines. Putrescine levels were more variable, however, trended toward decreased concentration in all cell lines and were significantly decreased in Saos-2 cells. Spermine levels were essentially unchanged, which is not uncommon and found also after DFMO treatment of neuroblastoma and other tumor cell lines [16]. Data represents three independent experiments (*N* = 3). * denotes statistically significant changes compared with control (*p* < 0.05).

To study the effect of DFMO on OS cell morphology and viability, OS cells were plated in 10-cm dishes and exposed to DFMO for six days. By the end of the exposure, treated cells were significantly less confluent, which was most evident with the MG-63 and U-2 OS cell lines (Figure 1A). This was not associated with morphologic changes seen with apoptosis. When viable cells were counted with a hemocytometer and trypan blue, the cell numbers in the treated samples were significantly reduced compared to control samples (Figure 1B). The half maximal inhibitory concentration (IC-50) values at 72 h were determined at 4.43 ± 1.19 mM (MG-63), 4.78 ± 1.41 mM (U-2 OS), and 5.14 ± 1.12 mM (Supplementary Figure S2).

### 3.2. Alpha-difluoromethylornithine Treatment Decreases Intracellular Polyamine Levels

To evaluate the effect of DFMO on intracellular polyamine levels, OS cells were exposed to DFMO for six days after which levels of putrescine, spermidine and spermine were measured by HPLC. In all cell lines there was a consistent decrease in putrescine and spermidine compared to controls. Changes in spermine were minimal. Although there was some variability in the change of putrescine in MG-63 cells, there was a clear overall trend toward decreased putrescine levels (Figure 1C–E). This is the same pattern seen in neuroblastoma [16] and other cancer models in which putrescine and spermidine are the two PAs most affected by DFMO, whereas spermine typically does not significantly change.

### 3.3. Alpha-difluoromethylornithine Induces Cell Cycle Arrest

**Figure 2:**
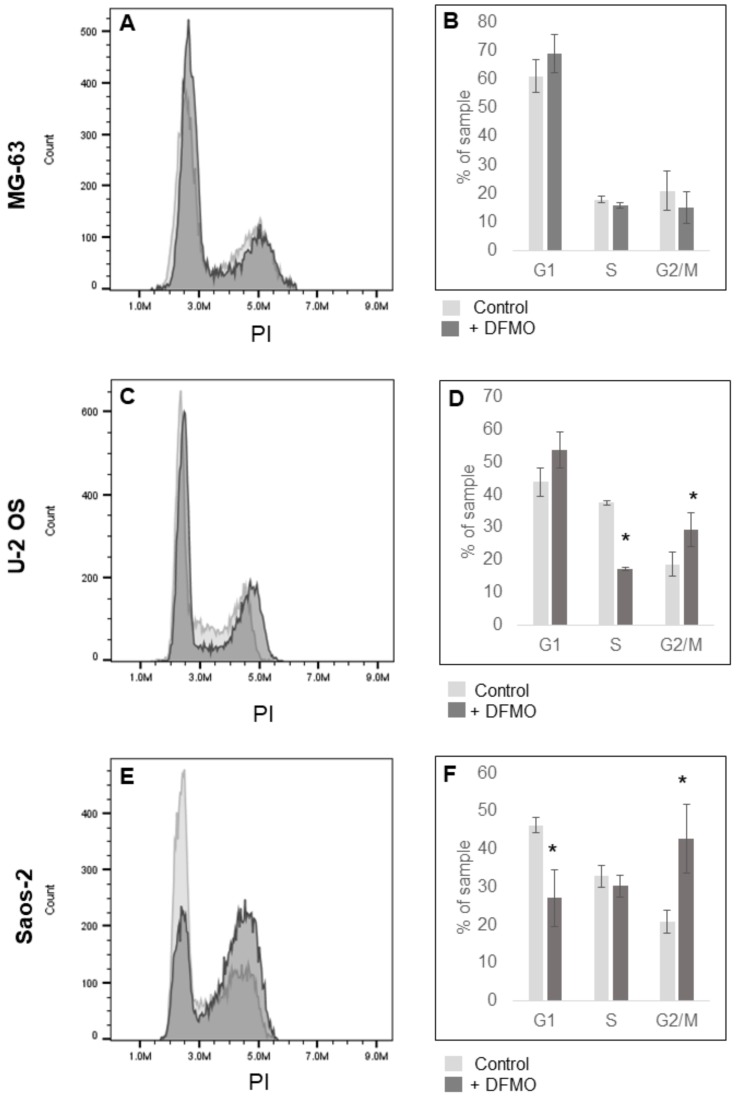
Effects of DFMO on cell cycle phase distribution in human OS cells. (**A**,**C**,**E**) Representative flow cytometry cell cycle analysis of MG-63, U-2 OS and Saos-2 cell lines stained with propidium iodide (PI) after 6 days of 5 mM DFMO exposure. Images are representative of three independent experiments (*N* = 3). (**B**,**D**,**F**) Percentage of cells in each phase of cell cycle averaged over at least three serial experiments (*N* = 3). U-2 OS and Saos-2 demonstrate G/2M cell cycle arrest after DFMO exposure. * denotes statistically significant changes in cell proliferation compared with control (*p* < 0.05).

Cells treated with DFMO did not appear apoptotic, suggesting that their decreased confluency was secondary to decreased proliferation rather than cell death. This prompted cell cycle analysis with flow cytometry after a six-day treatment with DFMO. Saos-2 cells showed profound G_2_/M cell cycle arrest with the percentage of cells in this phase increasing from 21% to 43% while the cells in G_1_ nearly halved from 46% to 27% (Figure 2E,F). U-2 OS cells showed combined G_1_ and G_2_/M cell cycle arrest with cells in G_2_/M increasing from 19% to 29% and cells in G_1_ increasing from 44% to 54% (Figure 2C,D). Thus it appeared that cell cycle arrest was, at least in part, the reason for the decreased cell proliferation in Saos-2 and U-2 OS. No cell cycle arrest was evident in MG-63 (Figure 2A,B).

### 3.4. Alpha-difluoromethylornithine Induces Differentiation

**Figure 3:**
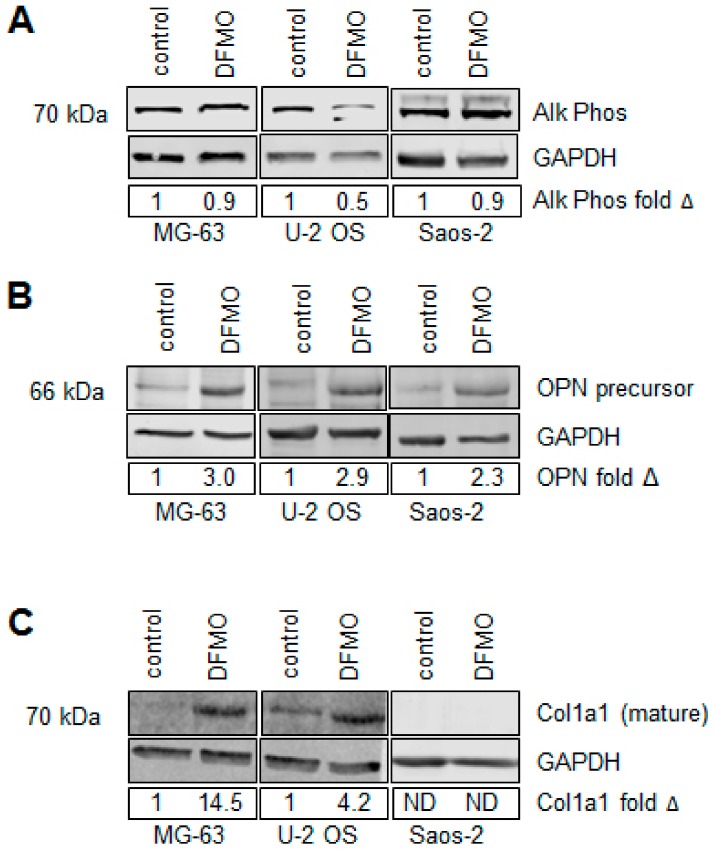
Differentiation of DFMO-treated human OS cells. Effect on the early differentiation marker (**A**) Alkaline phosphatase (Alk Phos) and late differentiation markers (**B**) osteopontin (OPN) and (**C**) collagen, type 1, alpha 1 (Col1a1), after six days of exposure to 5 mM DFMO. All cell lines showed increased levels of OPN, and MG-63 and U-2 OS displayed increased expression of Col1a1, demonstrating terminal osteogenic differentiation. Fold changes in protein levels (indicated below each blot) represent the average of quantified Western blot images from three independent experiments (*N* = 3).

To evaluate an alternative mechanism by which osteosarcoma cells could have decreased proliferation without undergoing cell cycle arrest, we evaluated markers of osteogenic differentiation after exposure to DFMO for six days. Cells were grown in standard RPMI media throughout the experiment. After exposure to DFMO, Western blot was used to measure alkaline phosphatase (Alk Phos), collagen 1a1 (Col1a1) and osteopontin (OPN). Alk Phos is an early osteogenic differentiation marker whereas OPN and Col1a1 are late osteogenic differentiation markers. Alk Phos protein levels did not increase in any of our tested cell lines (Figure 3A) whereas the OPN precursor protein levels consistently increased in the presence of DFMO treatment in MG-63, U-2 OS and Saos-2 cells (Figure 3B). In addition, Col1a1 protein levels were increased in MG-63 and U-2 OS cells (Figure 3C). These results indicate that DFMO treatment results in terminal differentiation of some osteosarcoma cell lines. Remarkably, this occurred even in the absence of osteogenic differentiation media.

### 3.5. Cell Recovery is Delayed by Alpha-difluoromethylornithine 

**Figure 4:**
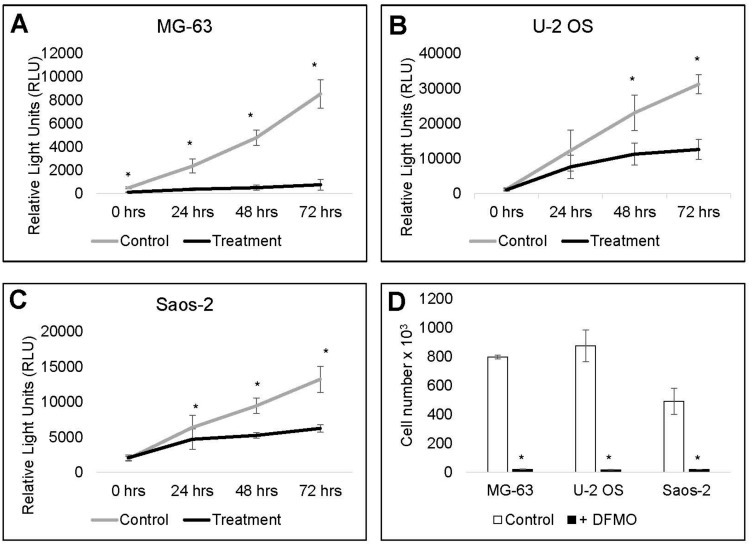
DFMO delays cell recovery in human OS cells. MG-63, U-2 OS and Saos-2 cells were exposed to 5 mM DFMO for six days and then reseeded in standard media. (**A**–**C**) Effect of treatment on cell recovery, 24, 48 and 72 h after removal of DFMO. Quantification of cell viability was determined by measuring relative light units (RLU) after the addition of RealTime-Glo^TM^ MT Viability assay reagent. Data represent three independent experiments done in triplicate (*N* = 9). Error bars represent standard deviation. MG-63 (**A**), U-2 OS (**B**) and Saos-2 (**C**) cell viability was markedly decreased after DFMO exposure; (**D**) to evaluate cell recovery beyond the 72 h time point, treated cells (six days with DFMO) or untreated control cells were washed, reseeded in standard media, and counted with a hemocytometer and trypan blue, seven days after DFMO removal. At this time point, previously treated cells still showed only minimal recovery compared to controls. Data represent three independent experiments done in triplicate (*N* = 9). Error bars represent standard error. * denotes statistically significant changes in cell proliferation compared with control (*p* < 0.05).

The differentiation driven by DFMO led us to evaluate whether the inhibition of cell proliferation would be sustained after removal of the drug. After six days, DFMO-treated cells and untreated control cells were washed and reseeded into 96-well plates. After being allowed to settle overnight, cell viability was measured at 24, 48 and 72 h. In all tested cell lines there was marked delay in recovery of DFMO-treated cells (Figure 4A–C). This was especially pronounced in MG-63, in which treated cells showed minimal growth change over 72 h. Due to assay limitations beyond the 72 h time point with the RealTime-Glo™ reagent, we performed a similar experiment using a hemocytometer and trypan blue to determine the cell viability seven days after DFMO was removed from cells. Strikingly, even seven days after DFMO removal, the treated cells showed only minimal recovery (Figure 4D), clearly suggesting that DFMO prolongs cell growth inhibition, even in the absence of the drug.

## 4. Discussion

Osteosarcoma is the most common childhood bone tumor. In patients with metastatic or refractory disease, overall survival is only around 30% despite aggressive chemotherapy and surgical resection. In the last three decades there have been no significant developments that have improved survival, and new therapies are greatly needed. An important aspect of treatment both upfront and at relapse is to get a patient into a complete remission (CR) with chemotherapy and resection such that there is no visible evidence of disease. In the setting of relapse this may not provide a cure, however, it can extend life for years before another relapse occurs. Presumably then, there are residual, unmeasurably small amounts of viable osteosarcoma cells after completion of therapy which later reestablish themselves leading to recurrence. Hence, a prophylactic or maintenance regimen that could cause these cells to differentiate and lose their stem cell potential would prevent relapses. An oral medication with few adverse effects that could potentially be taken for long periods of time would be ideal. DFMO would be just such a treatment. Further, though DFMO does not seem to have much effect as an adjunct to upfront aggressive therapy in several cancer models, it has been shown to be effective at preventing cancer development in patients at high risk of developing primary cancer [19,20,21,24] or recurrence [24,25,28].

c-MYC is a direct transcriptional activator of the *ODC1* gene, which leads to ODC overexpression and PA-dependent cell proliferation. Our data demonstrate that the ODC inhibitor DFMO suppresses cell proliferation in three OS cell lines which express c-MYC and ODC (Supplementary Figure S1), through a combination of processes including cell cycle arrest and differentiation. The DFMO-mediated induction of differentiation has previously been observed in Friend’s murine erythroleukemia (MEL) cells [34]. Although early differentiation markers were unchanged, those that are seen later in osteogenic maturation were increased with DFMO treatment, demonstrating that DFMO leads to terminal differentiation of these OS cell lines. When differentiation has been evaluated previously as a potential treatment target of OS therapy, there have been concerning results. Potent osteogenic bone morphogenetic proteins (BMPs) used to induce differentiation in some of these same cell lines actually resulted in increased proliferation in vitro and increased tumor growth in orthotopic in vivo models. The hypothesized mechanism was that differentiation defects in human OS cells only allow them to reach an early progenitor stage that is stuck in an early proliferative phase. This results in increased proliferation when exposed to osteogenic stimuli. However, when later steps of the differentiation pathway were induced, these cells were able to differentiate and they showed decreased proliferative activity [35]. Our results suggest that PA inhibition activates later steps of the differentiation pathway and pushes OS cells through the early progenitor stage to terminal differentiation, which results in decreased proliferation.

Furthermore, the effects of DFMO on OS cell growth remain for at least seven days after removal of DFMO. When OS cells exposed to DFMO for six days were reseeded in standard media, they continued to have astonishingly depressed growth for up to seven days. ODC is a rapidly metabolized enzyme; thus, once DFMO is removed, functional ODC and PAs quickly re-accumulate. The prolonged effects of DFMO on cell proliferation suggest that it might also induce effects independent of PA depletion. 

From current clinical trials we have learned that extended treatment with DFMO in children is feasible and well tolerated [28]. The prolonged effects of DFMO exposure as observed in this study are particularly encouraging for progression into OS animal models and future clinical trials, as it suggests that even when there are times during which DFMO is not at a therapeutic level, there will be continued effects. This may also justify an easier, less frequent dosing schedule.

## 5. Conclusions

In conclusion, our results demonstrate that inhibition of PA synthesis with DFMO induces cell cycle arrest and terminal differentiation in human OS cells. These effects are persistent even after removal of DFMO. These findings suggest a role for DFMO in OS therapy, particularly in the setting of preventing relapse. Even when patients have achieved a radiographic complete remission with aggressive chemotherapy and surgical resection, at times, low levels of viable OS cells remain, resulting in later relapse. If these residual cells could be pushed into cell cycle arrest or toward terminal differentiation, relapse could potentially be prevented or delayed. Thus, further studies are needed to determine whether our results persist in vivo in OS mouse models.

## Supplementary Materials

The following are available online at http://www.mdpi.com/2076-3271/6/3/65/s1. Figure S1: c-MYC and ODC1 expression in OS cell lines, Figure S2: DFMO IC-50 in OS cell lines.
