# Supplementary material for: Polyamine Biosynthetic Pathway as a Drug Target for Osteosarcoma Therapy
Source: Med Sci (Basel). 2018 Aug 16;6(3):65. doi: 10.3390/medsci6030065 (PMC6165283; doi:10.3390/medsci6030065)
Supplement: Supplementary file 1 [file medsci-06-00065-s001.pdf]

Bio-informatic Analysis

The Versteeg-86 dataset was queried using R2, the genomics analysis and visualization platform developed by Jan Koster in the Department of Oncogenomics at the Amsterdam University Medical Center, University of Amsterdam (http://r2.amc.nl). Expression data (CEL files) were analyzed as described in [12]. Briefly, gene transcript levels were determined from data image files using GeneChip operating software (MAS5.0 and GCOS1.0, from Affymetrix, Santa Clara, CA, USA). Samples were scaled by setting the average intensity of the middle 96 % of all probe-set signals to a fixed value of 100 for every sample in the dataset, allowing comparisons between micro-arrays. The TranscriptView genomic analysis and visualization tool within R2 was used to check if probe-sets had a unique anti-sense position in a late coding exon or the 3′ UTR of the gene (http://r2.amc.nl > genome browser). The probe-sets selected, 202431_s_at and 200790_at, meet all these criteria and showed the highest expression in Versteeg-86 for c-MYC and ODC1, respectively. Graphs show 2log (Panel A), or normal (Panel C) expression according to MAS5.0: values >1000 represent high-level expression. All expression values and other details for the datasets can be obtained through the R2 website.

Alpha-difluoromethylornithine IC-50 Determination

Osteosarcoma (OS) cell lines were plated in transparent flat 96-well plates overnight. The cells were exposed to different alpha-difluoromethylornithine (DFMO) concentrations (0 – 25 mM) for 72 hours. MG-63 and U-2 OS cell viability was determined using Cell Titer 96 Aqueous One Solution Cell Proliferation Assay (Promega, Madison, WI, USA) according to the manufacturer's protocol. Briefly, after the 72 hour treatment, 20 µl of reagent was added to each well and incubated at 37 ˚C for 3 hours. Absorbance was then measured at 490 nm using a Biotek Synergy microplate reader (Winooski, VT, USA). Saos-2 cell viability was determined using Sulforhodamine B (SRB). After treatment, cells were fixed with 10% trichloroacetic acid (TCA) for one hour at 4˚ C. Cells were washed three times with water, air dried, and stained with 100 µl 0.4% SRB in 1% acetic acid for 20 minutes. Excess SRB was removed by washing with 1% acetic acid five times. After drying, 100 µl of 10 mM Tris-HCl (pH 7.0) was added to each well and the plates were shaken for 10 minutes. Absorbance was then read at 540 nm using a Biotek Synergy microplate reader (Winooski, VT, USA). For all the cell lines, cell viability at the initiation of treatment (T=0) was measured and subtracted from the 72 hour values. IC-50 curves and values were determined using Graphpad Prism 5 software (La Jolla, CA, USA).

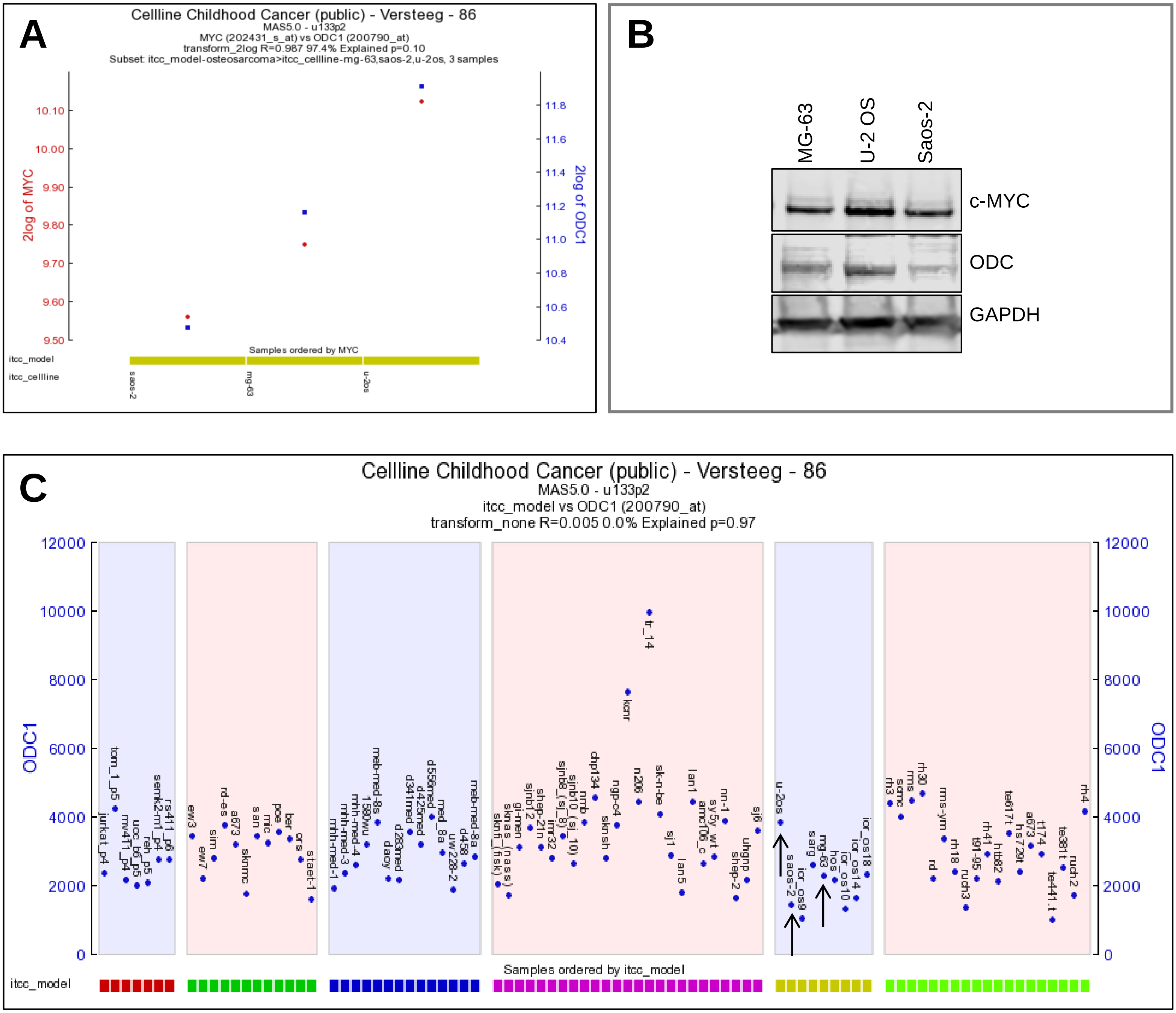

Supplementary Figure 1. c-MYC and ODC1 expression in osteosarcoma (OS) cell lines. **(A) c-**MYC and ODC1 mRNA expression for the OS cell lines in this study, according to Versteeg-86, the largest public mRNA profiling dataset for pediatric cancer cell lines. The graph shows that c-MYC and ODC1 are robustly expressed in the Saos-2, MG-63, and U-2 OS cell lines, and that ODC1 expression closely follows c-MYC expression, suggestive of ODC1 regulation by c-MYC. **(B)** c-MYC and ODC protein expression in osteosarcoma cell lines six days after plating. Similar to the mRNA expression profiles, higher c-MYC protein expression correlated with higher ODC expression. The Western blot images shown are representative of three independent experiments (n=3). **(C)** ODC1 mRNA expression for all pediatric cancer cell lines in Versteeg-86. The graph shows that all 86 pediatric cell lines express high ODC1 mRNA levels. OS cell lines (yellow bars) have ODC1 expression comparable to other major pediatric cancers like acute lymphoblastic leukemia (red bars), Ewing sarcoma (green bars), medulloblastoma (blue bars), neuroblastoma (maroon bars), and rhabdomyosarcoma (light green bars). The graph also shows that the Saos-2, MG-63, and U-2 OS cell lines are representative of low, medium, and high ODC1 expression in OS cells *in vitro*, respectively.

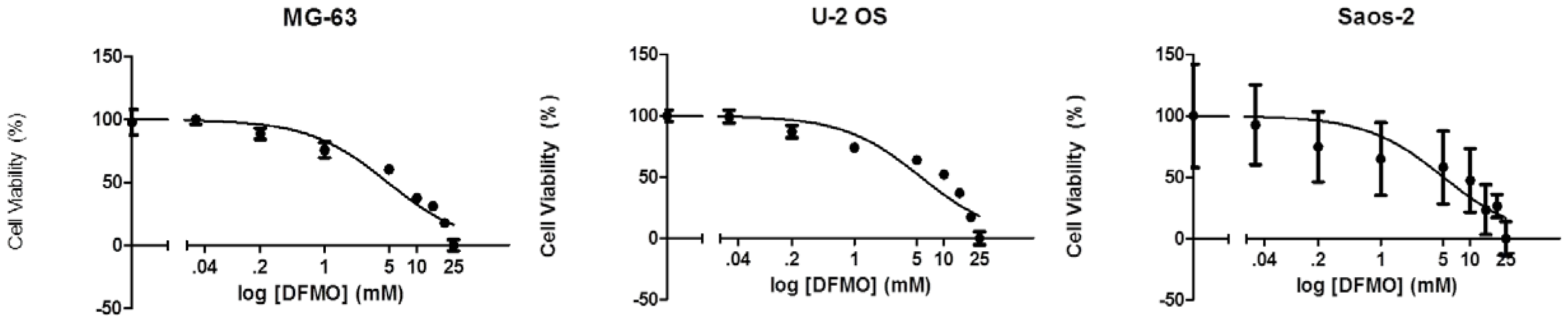

IC-50 (mM): 4.43 +/- 1.19 | 4.78 +/- 1.41 | 5.14 +/- 1.12

Supplementary Figure 2. Alpha-difluoromethylornithine (DFMO) IC-50 in osteosarcoma (OS) cell lines. OS cell lines were exposed to increasing concentrations of DFMO (0 to 25 mM) for 72 hours. Cell viability was determined using Cell Titer 96 Aqueous One Solution Cell Proliferation Assay (Promega) for MG-63 and U-2 OS. Saos-2 cell viability was determined using Sulforhodamine B (SRB). IC-50 curves and values were generated using Graphpad Prism 5. Data represents three independent experiments done in triplicate (N=9).
